# The cervicovaginal microbiome: an emerging determinant of HPV infection and disease

**DOI:** 10.1128/jvi.01706-25

**Published:** 2026-07-23

**Authors:** Megan E. Spurgeon

**Affiliations:** 1 John W. and Jeanne M. Rowe Center for Research in Virology, Morgridge Institute for Research 145254, Madison, Wisconsin, USA; 2 McArdle Laboratory for Cancer Research, Department of Oncology, School of Medicine and Public Health, University of Wisconsin-Madison 5228, Madison, Wisconsin, USA; 3 University of Wisconsin Carbone Cancer Center 206022, Madison, Wisconsin, USA; Universiteit Gent, Merelbeke, Belgium

**Keywords:** human papillomavirus, microbiome, viral pathogenesis, cervical cancer, murine papillomavirus

## Abstract

Human papillomaviruses (HPVs) are epitheliotropic viruses that cause diseases ranging from cutaneous warts to invasive cancers. HPVs exhibit tropism for the epithelial lining of the skin, oral cavity, and anogenital tract. A subset of HPVs, known as high-risk HPVs, have oncogenic potential and cause nearly one-third of all virus-associated cancers worldwide, including essentially all cervical cancers, most other anogenital cancers, and a growing percentage of head and neck cancers. A large and growing body of clinical and epidemiological research has established the host microbiome, or the collection of microorganisms on and within the body, as a key determinant of HPV infection, persistence, and disease development. In this review, an overview of the current understanding of associations between bacterial components of the host cervicovaginal microbiome (CVM) and aspects of HPV pathogenesis is discussed. Potential mechanisms through which the CVM influences HPV infection and neoplastic disease development are also presented. The inherent challenges of studying HPVs and the microbiome have impeded progress to define not only the underlying mechanisms involved in HPV-CVM interactions, but also the ability to address remaining questions such as causality, temporal dynamics, and the directionality of this relationship. Emerging *in vitro* and *in vivo* models provide powerful opportunities to address these outstanding questions and increase our understanding of trans-kingdom interactions that contribute to HPV infection and subsequent disease.

## INTRODUCTION

Viruses are obligate intracellular parasites that depend on their hosts to replicate. A productive outcome of viral infection is therefore heavily dependent upon the host conditions that viral particles, genomes, and gene products encounter at steps throughout the viral life cycle. Factors that can influence the successful replication of viruses include the natural flora present in target tissues and cells, metabolic activity that sustains cell growth, functional transcriptional and translational machinery, and the activity of signaling pathways. Together, these conditions create an environment that is either conducive or detrimental to viral replication and progeny virion production. The study of these host conditions and their collective impact on viruses will not only better our understanding of viral pathogenesis but may also help reveal new therapeutic vulnerabilities.

An important host factor increasingly recognized for its effects on human health is the microbiome, a term that describes the ecological niche of microorganisms present within and on the body. The microbiome is composed of both commensal and/or pathogenic microbiota, including bacteria, yeast, fungi, and viruses. It often adopts unique and dynamic compositions across anatomical sites and pathological conditions (1). Collectively, microbiota are estimated to comprise at least half of all cells in the human body, with the remaining cells being of human origin (2, 3). These microbial communities exert such a significant influence on tissue homeostasis and function that the microbiome has been referred to as the “second genome” of the human body, affecting various aspects of human health and the etiology of disease (4, 5). Considering its pervasive impact on human physiology, the microbial milieu encountered during viral infection likely plays a significant role in shaping viral pathogenesis, transmission, and disease.

A growing body of evidence suggests that the microbiome is a key determinant of human papillomavirus (HPV) pathogenesis (6, 7). HPVs are ubiquitous DNA viruses that cause a range of diseases in humans spanning from benign warts to invasive cancer. Thus far, most associations between the microbiome and HPVs have been demonstrated almost exclusively through clinical and epidemiological correlations determined by comparing aspects of viral infection/disease and corresponding microbial community structures. The lack of mechanistic data needed to establish a causal relationship between microbiota and HPV pathogenesis can be explained, at least in part, by the challenges of HPV and microbiome research. This review discusses the current state of knowledge regarding the associations between papillomaviruses and the microbiome, with a focus on this relationship in the cervical and vaginal (cervicovaginal) epithelium, a common site of HPV infection and disease. The emergence of new and developing models that recapitulate cervicovaginal microbiome dynamics and the intricacies of the HPV life cycle are valuable tools that will help define the causal and temporal relationships between microbiota and HPVs.

## BACKGROUND

### Papillomaviruses: tropism and life cycle

**Fig 1:**
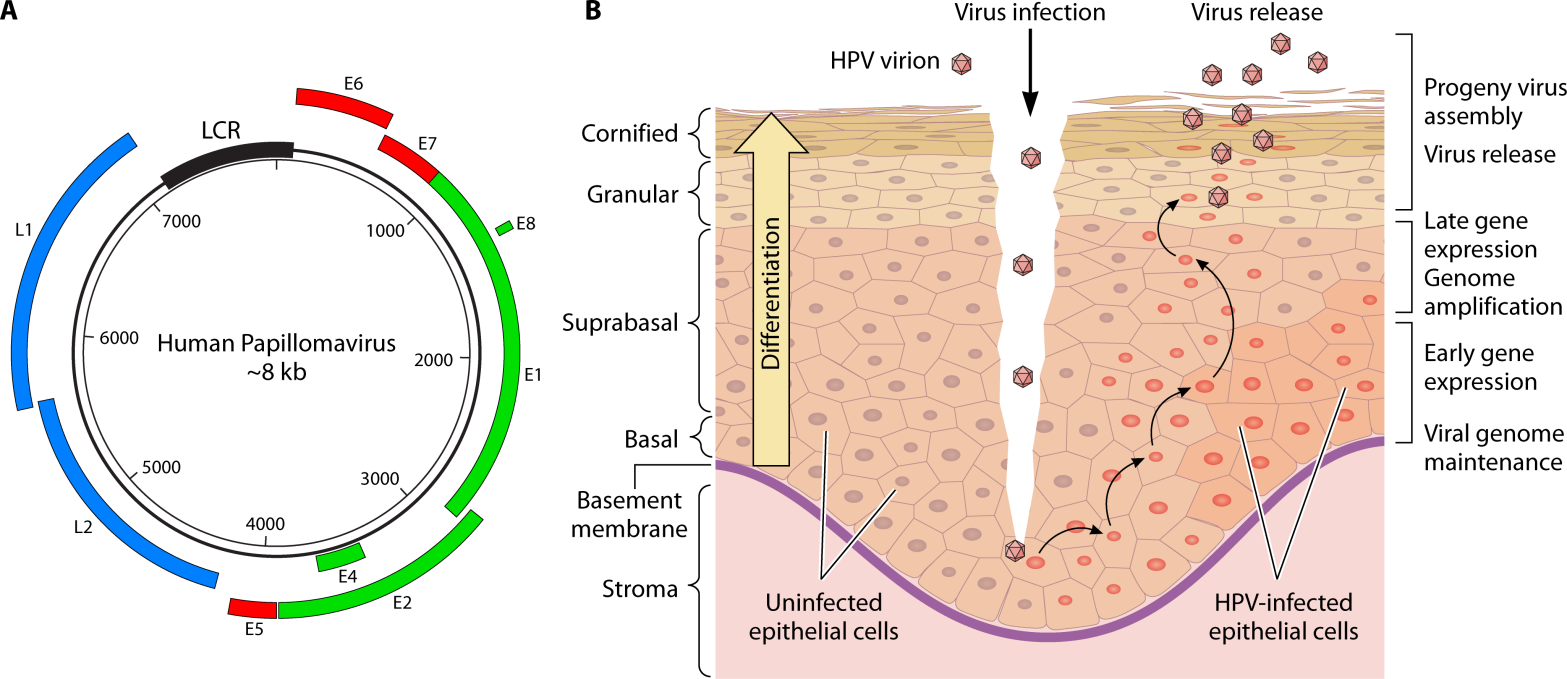
The HPV genome and life cycle. (**A**) The human papillomavirus genome is a circular, double-stranded DNA episome approximately ~ 8 kilobases (kb) in length. The genome is organized into early (E) and late (L) regions that are temporally expressed during the viral life cycle. Oncoproteins expressed by high-risk HPVs are shown in red. Other early genes are shown in green, and late genes are shown in blue. LCR, long control region. (**B**) Overview of the HPV life cycle in stratified squamous epithelium. Layers of the epithelium (left) and key events during the virus life cycle and their spatial distribution (right) are indicated.

*Papillomaviridae* is a large and diverse family of non-enveloped, icosahedral viruses that are highly species-specific and infect a broad range of hosts, including reptiles, fish, birds, and mammals (8, 9). Papillomavirus virions are small (~55 nm) and contain a single copy of a circular, double-stranded DNA genome between 5 and 8 kilobases in size (Fig. 1A). The archetypal HPV genome is composed of early and late regions that typically encode eight open reading frames, although there are slight variations among different virus types. “Early” (E) and “late” (L) viral gene expression occurs in a temporal fashion and is regulated by a complex pattern of promoter use and splicing (10). Papillomavirus early genes (E1, E2, E4, E5, E6, and E7) have various functions that support viral DNA replication, host cell proliferation, and immune evasion, while the late genes (L1 and L2) are structural proteins necessary for progeny virion capsid formation. Papillomaviruses are epitheliotropic viruses, meaning they exhibit strict tissue tropism for the stratified squamous epithelium, and can productively infect both cutaneous (keratinized) and mucosal epithelia. In humans, various anatomical sites lined with stratified epithelial tissue are susceptible to HPV infection, including the skin, oral cavity, upper respiratory tract, and several anogenital tissues such as the cervix, vagina, vulva, penis, and anus. The stratified epithelium functions as a protective barrier at these sites, with its architecture and physiology strictly controlled by a process of terminal cellular differentiation that yields several distinct layers of cells with different proliferative capacities and programs of gene expression.

The papillomavirus life cycle is intimately linked to and dependent on this tightly controlled process of epithelial differentiation (11, 12) (Fig. 1B). Papillomaviruses infect undifferentiated basal keratinocytes present in the bottom layer of the stratified epithelium. Virion access requires microwounds that compromise the integrity of the epithelial barrier. Basal keratinocytes are the only cells within the stratified epithelium that actively proliferate and divide, essential activities for papillomavirus genome nuclear entry and maintenance in host cells (13). Papillomavirus virions bind heparin sulfate proteoglycans on the basement membrane, prompting conformational changes and cleavage events that facilitate internalization and subsequent intracellular trafficking of the viral capsid (14). Once established, the circular viral genomes, or episomes, are maintained at low viral copy numbers in basal cells. Upon cell division of an infected basal cell, daughter cells begin to undergo terminal differentiation toward the apical surface of the epithelium. During this phase of infection, parabasal and suprabasal cells are reprogrammed by early viral genes to proliferate, thus allowing viral DNA replication to occur in additional layers of the epithelium. The number of viral genomes is significantly increased during differentiation in a process called viral genome amplification, which is dependent on host DNA replication and repair machinery (15–17). The accumulation of viral genomes and the synthesis of late viral capsid proteins in the uppermost layers of the stratified epithelium support the production of progeny virions, which are released from terminally differentiated cells that slough from the apical tissue surface. For this reason, HPVs are transmitted through direct contact between epithelial tissues, such as skin-on-skin contact or through anogenital contact during sexual activity. In humans, the full HPV life cycle takes 2–3 weeks to complete (18). The intricate spatiotemporal dependence of the papillomavirus life cycle on cellular differentiation helps explain their tissue tropism but also introduces several opportunities for regulation by intrinsic and extrinsic factors that influence epithelial homeostasis.

### Human papillomaviruses: etiological agents of human disease and cancer

There are currently more than 200 papillomaviruses known to infect humans (19). HPVs are informally classified by their tropism for either mucosal or cutaneous epithelia. Most of the mucosotropic HPVs that infect the cervix, vagina, oral cavity, and other anogenital sites belong to the *Alphapapillomavirus* genus, whereas the cutaneotropic HPV types that infect the skin belong to the *Betapapillomavirus* and *Gammapapillomavirus* genera (9). The ~40 HPV types that infect the anogenital epithelia are the most common sexually transmitted infection (STI) worldwide, with estimates suggesting that more than 80% of sexually active individuals are infected at least once during their lifetime (20–23).

HPVs are further classified by their oncogenic potential, or risk of causing cancer. Low-risk *Alphapapillomavirus* types, such as HPV6 and HPV11, are most frequently associated with benign genital warts and recurrent respiratory papillomatosis. On the other hand, high-risk *Alphapapillomavirus* HPVs are causal agents of anogenital cancers (cervix, vagina, vulva, penis, and anus) and oropharyngeal cancers (24). Two high-risk HPVs, HPV16 and HPV18, cause most cancers across anatomical sites (25–28). *Betapapillomavirus* infections cause benign skin warts, or papillomas, and can cause non-melanoma skin cancers and squamous cell carcinomas (SCCs) in patients with certain genetic and/or environmental conditions (29). Considering their ubiquitous prevalence and etiological links to human malignancies, HPVs remain a significant global health burden despite the availability of highly effective prophylactic vaccines (30).

High-risk HPVs are one of seven known human tumor viruses that together cause at least 15% of human cancers worldwide (31). Oncogenic HPVs alone are the etiological agents of one-third, or ~5%, of virus-induced cancers globally (32). However, cancer is an atypical and aberrant outcome of HPV infection. Although most anogenital infections are cleared and do not progress to invasive cancer (33–35), HPVs can establish decades-long or even lifelong persistent infections. Persistent HPV infections, type, and viral load are some of the strongest predictors of progression to cancer in HPV-infected individuals (35–40). Persistent high-risk HPV infections can result in integration of the viral genome into host DNA, a dead end to the productive viral life cycle. As precancerous lesions progress to invasive cancer, the number of cells with integrated genome increases (41). Integration confers a growth advantage and leads to higher and more stable expression of the viral E6 and E7 oncogenes, frequently through loss of the repressive functions of the viral E2 protein (42) and/or the integrated genome acting as a transcriptional super-enhancer (43, 44). Despite these cancer-promoting activities, HPV viral genome integration is not a prerequisite for carcinogenesis and can vary substantially in cancers depending on HPV type and anatomical site (45–47).

The high-risk HPV E6 and E7 proteins are highly multifunctional oncoproteins that drive cellular transformation, neoplastic disease, and carcinogenesis (48, 49). The primary oncogenic mechanisms of E6 and E7 are inactivation of p53 and retinoblastoma protein (Rb), respectively, two major host tumor suppressors that control the cell cycle and cellular proliferation (50). The oncogenic potential of high-risk HPVs can be explained, at least in part, by functions specific to their oncoproteins. For instance, low-risk E7 proteins have a much weaker binding affinity for Rb than high-risk E7 (51, 52), and low-risk HPV E6 and E7 proteins have significantly reduced capacity to immortalize and transform cells (53, 54). The high-risk HPV oncoproteins have several additional established and/or predicted oncogenic mechanisms such as manipulation of epithelial differentiation, genome instability, epigenetic alterations, and influences on every hallmark of cancer (55–57). However, HPV infection alone is not sufficient for carcinogenesis, suggesting contributions from other co-factors are involved (58).

### The cervicovaginal microbiome and dysbiosis

The microorganisms that colonize the female reproductive tract are commonly referred to as the vaginal microbiota and the resulting milieu and conditions referred to as the vaginal microbiome. Anatomically, these terms often represent not only the epithelial lining of the vagina, but also the cervix. Therefore, “cervicovaginal microbiome” (CVM) is a more appropriate descriptor of the microbiome associated with HPV infections. Cervicovaginal health is influenced by bacterial species (“bacteriome”), fungi (“mycobiome”), and viruses (“virome”) that comprise the CVM (59–61). Because they are significantly more characterized and well-studied than other microbiota, this review will focus on bacterial components of the CVM as they relate to HPV pathogenesis and disease.

The current definition of an archetypal “healthy” CVM is one that is dominated by one or more *Lactobacillus* species (spp.) (62–64). *Lactobacillus* spp. produce lactic acid and other antimicrobial compounds that help maintain a low pH (less than 4.5), prevent colonization of pathogenic microbes, and support homeostasis in the cervicovaginal tract (65–68). However, as the pool of women whose CVMs are characterized continues to expand and diversify, “healthy” CVMs exist across a broad spectrum and can differ by variables such as geographical location, stress, ethnicity, and socioeconomic status (69–71). To classify CVM compositions, high-throughput sequencing data from cross-sectional and longitudinal studies of over 400 healthy women were used to establish five community state types (CSTs) (63, 64). Approximately 75% of women have CVMs that fall within four CSTs characterized by low bacterial diversity and dominance by one or more different *Lactobacillus* spp. (CST I-*Lactobacillus crispatus*, CST II-*Lactobacillus gasseri*, CST III-*Lactobacillus iners*, and CST V-*Lactobacillus jensenii*). On the other hand, CST IV is characterized by high bacterial diversity of non-*Lactobacillus* spp., generally anaerobic bacteria. CST stability varies greatly in women, likely due to the influence of factors like hormones, diet, sexual activity, hygiene practices, and genetic factors (72). CST classification is a widely used way to describe CVM community compositions, although a downside is that it can obscure the presence and contributions of less abundant and/or rare bacterial taxa. Overall, the CVM is a dynamic community of microorganisms with a critical role in maintaining female reproductive tract health (62, 73). However, both intrinsic and extrinsic factors can alter the bacterial species normally present in an individual, leading to a state of imbalance called dysbiosis.

The most common form of cervicovaginal dysbiosis in reproductive-aged women is bacterial vaginosis (BV). Worldwide prevalence of BV is approximately 26%, although there are regional and ethnic differences (74). The main symptom of BV is vaginal discharge, which causes discomfort and negatively impacts quality of life. BV is difficult to treat and is highly recurrent in certain individuals (75, 76). There is strong evidence supporting sexual transmission of BV, which was further strengthened by the recent finding that male partner treatment reduces BV recurrence (76–78). The CVM of women with BV often closely mirrors conditions found in CST IV, including depletion of *Lactobacillus* spp. and a concomitant increase in the abundance of strict anaerobes like *Gardnerella vaginalis*, *Prevotella bivia*, *Sneathia* spp., and *Fannyhessea vaginae* that form a polymicrobial biofilm on the cervicovaginal epithelium (79, 80). The precise etiology of BV remains elusive, further complicating effective treatment options. Emerging models describe a process by which BV-associated bacteria (BVAB) coordinate biofilm development through an orchestration of primary colonizers that create metabolic and pathophysiological conditions suitable for the growth of secondary colonizers (77, 81–83). These BVAB-associated polymicrobial biofilms and corresponding reduction in *Lactobacilli* significantly affect the physiology and barrier function of the cervicovaginal epithelium through metabolic, inflammatory, and immune mechanisms (81, 84–89). BV is associated with adverse reproductive outcomes, such as miscarriage and low birth weight, pelvic inflammatory disease, and increased susceptibility to STIs (73, 77), including gonorrhea, chlamydia, human immunodeficiency virus (HIV), herpes simplex virus type 2 (HSV-2), and HPVs (90–93).

## MICROBIOME INFLUENCES ON HPV INFECTION AND PATHOGENESIS

At various sites in the human body, the microbiome is an important host factor that influences infection and pathogenesis with a variety of viruses, including HPVs (94). In this section, the process of HPV-mediated neoplastic disease will be described, followed by a discussion of the current knowledge about the associations between the CVM at different stages along this progressive process and potential mechanisms by which it may influence HPV-associated malignant progression. Notably, most of this knowledge is derived from clinical studies, meta-analyses, and various “omics” approaches, thus highlighting the need for tractable *in vitro* and *in vivo* models.

### Overview of HPV-associated cervicovaginal neoplastic disease and cancer

**Fig 2:**
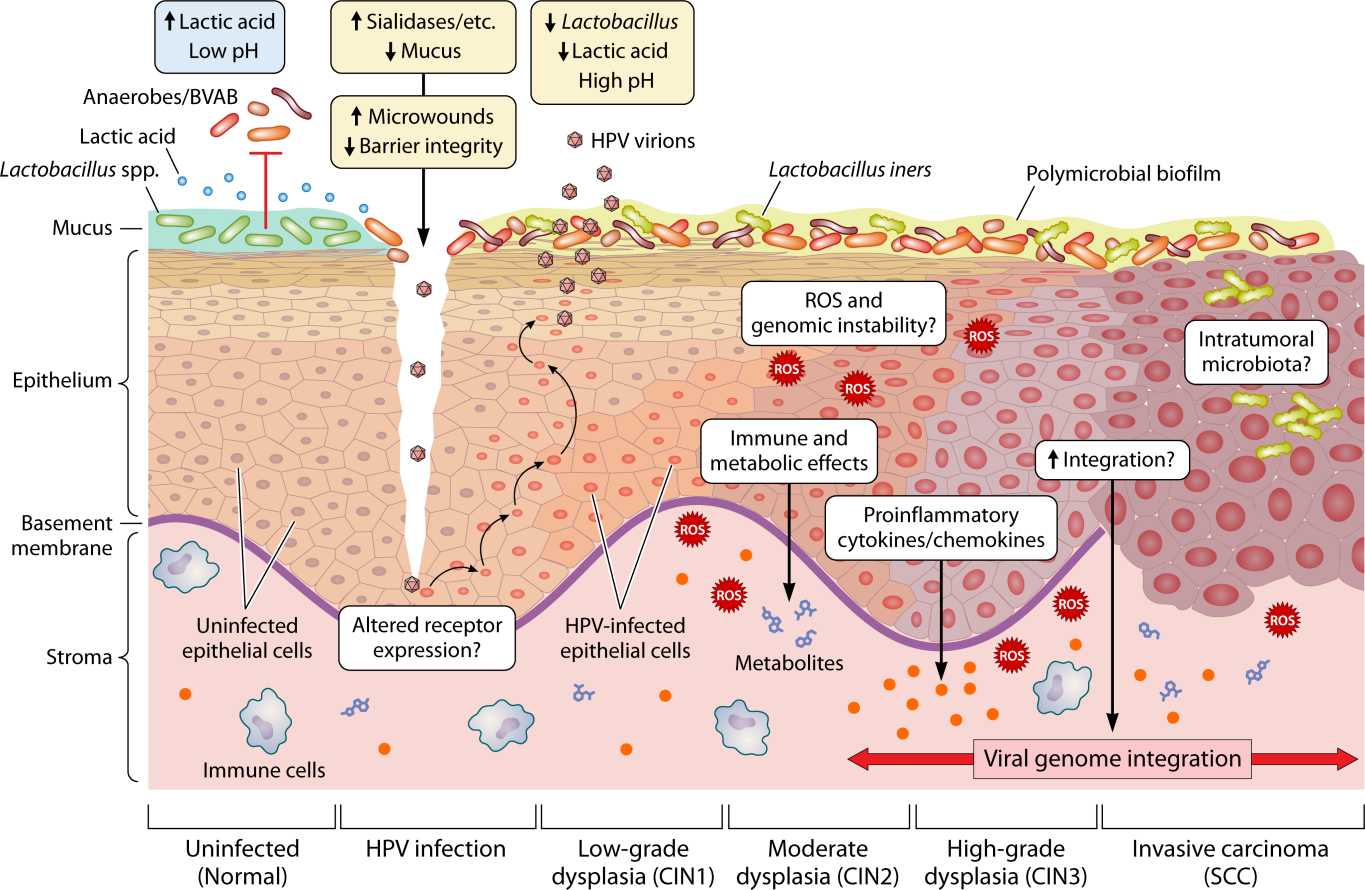
HPV-induced neoplastic disease and association with the cervicovaginal microbiome. An overview of the stages of HPV-induced neoplastic progression and potential aspects hypothesized as being influenced by cervicovaginal microbiota. Potential mechanisms and remaining questions are highlighted in boxes. Please refer to the text for additional discussion of these topics.

In the female reproductive tract, high-risk HPVs cause cancer through a progressive neoplastic disease process (12, 95–97) (Fig. 2). Usually, over the course of years or even decades, disease development progresses through stages of dysplasia with increasing severity called cervical intraepithelial neoplasia grades 1 through 3 (CIN1-3). Conventionally, low-grade CIN1 lesions support the HPV life cycle and produce progeny virions, and E6 and E7 oncoprotein expression remains low and comparable to that during productive infection. In most cases, CIN1 lesions regress and resolve within 2 years (98, 99). CIN2 lesions exhibit increased basal cell content and dysregulated cellular differentiation, which reduces or eliminates viral replication. Cells in CIN2 lesions have nuclear atypia and other traits of moderate dysplasia as well as highly elevated expression of E6 and E7. These characteristics continue to worsen in high-grade dysplastic lesions categorized as CIN3, which also begin to exhibit histological signs of chromosomal aneuploidy (100). It is unclear when viral genome integration occurs during neoplastic progression, but the current paradigm is that neoplastic progression to malignancy is an integration-driven event and occurs during the CIN2/CIN3 transition. Severely dysplastic CIN3 lesions have highly dysregulated E6 and E7 expression throughout multiple layers of the epithelium and increased proliferation, both of which are known consequences of integration (101). Notably, both episomal and integrated states of high-risk HPV genomes are found in cancers, with significant variations observed by HPV type and anatomical site (102, 103). The progression from CIN3 to invasive cervical cancer is likely a multifactorial event that stems from a combination of high E6 and E7 expression, alterations in key signaling pathways, and changes in the microenvironment (104–106). The high-risk HPV E6 and E7 oncoproteins have well-established roles in causing chromosome abnormalities, mitotic disturbances, and genomic instability, functions that are exacerbated by their dysregulation of DNA damage signaling and repair mechanisms (100, 107–111). Approximately 30% of untreated CIN3 lesions progress to invasive cervical cancer, although the molecular events that regulate this biological tipping point are not well-defined (112, 113). Epidemiological studies strongly support that HPV is necessary for CIN development and is the major risk factor for cervical cancer development, but these data also establish that HPV infection alone is insufficient (95).

### HPV infection and virus types

The first possible stage for the CVM to influence HPV cervicovaginal pathogenesis is during transmission and primary infection, a point when viral particles first interact with the cervicovaginal epithelial lining (Fig. 2). The conditions at this interface conceivably regulate whether, when, and to what extent a particle initiates a successful infection. The logistical complexity of capturing samples specifically during transmission with any level of temporal precision has limited our ability to define CVM associations during this stage of infection. However, Brotman and colleagues longitudinally tracked sexually active women over the course of 16 weeks, during which time the women collected self-samples twice a week that were analyzed by 16S ribosomal RNA (rRNA) sequencing to evaluate CVM composition and HPV testing to track infection (114). A transition from a negative to positive HPV test was highly correlated with CVM community structures dominated by either *Lactobacillus iners* (CST-III) or those with low *Lactobacillus* and high *Atopobium* (CST-IV). Notably, CVM composition was not associated with acquisition of HPV but correlated with remission or clearance of HPV. In a Korean female twin study by Lee et al., the CVMs of twins with discordant HPV infection status (one negative, one positive) were compared (115). Like women in the Brotman study, the CVMs of HPV-positive twins were associated with decreased *Lactobacillus* spp., presence of *Sneathia* spp., and higher diversity. More dynamic and unstable CVMs, as well as BV, are also linked to increased acquisition of HPV16 (116, 117). Multiple meta-analyses also support positive associations between cervicovaginal dysbiosis or BV with increased risk for HPV infection (93, 118), and the presence of *Lactobacillus* spp. correlates with decreased detection of high-risk HPV infections (119). However, some analyses contradict these findings, showing no association between BV and HPV infection (120, 121). Taken together, there is strong evidence suggesting that the composition of the host CVM influences HPV infection, but some data challenge this association.

Emerging data suggest that the HPV-CVM interaction may be virus type-specific. For example, Norenhag and colleagues found no significant difference in the CVM composition between HPV-negative women and women with low-risk HPV infections, but CVMs of women with high-risk HPV infections were significantly associated with anaerobic BVAB, including *Fannyhessea vaginae* and *Gardnerella vaginalis* (122). A recent study also found that HPV16 and HPV18, the two high-risk types associated with most HPV-associated cancers (123), are associated with different CVMs than other high-risk types and that HPV16, in particular, was most associated with dysbiosis (124). Future studies to address HPV type-specific associations will help identify microbial community compositions associated specifically with oncogenic HPVs, perhaps helping to illuminate host-pathogen interactions that contribute to anogenital disease and cancer.

### HPV persistence and clearance

Once a primary HPV infection is established, there are two possible outcomes: viral clearance and viral persistence. Incident cervicovaginal HPV infections generally last several months, and the duration, on average, is longer in high-risk compared to low-risk HPV types (125). Ultimately, around 90% of HPV infections are cleared by immune surveillance (34, 35). Alternatively, HPVs can establish long-lived persistent infections that evade host immunity (126). The precise factors that dictate clearance versus persistence remain unclear, although multiple groups have demonstrated that CVM composition is a strong determinant.

A large meta-analysis that exclusively evaluated longitudinal, prospective studies involving a total of over 100,000 women found that those with cervicovaginal dysbiosis were more prone to HPV persistence (118). More specifically, there are strong associations between persistent HPV infections and BV and/or BV-related CVMs. In a study that longitudinally tracked over 400 women with high-risk HPV infections, the prevalence of BV in women with persistent infections was more than double that of women who cleared the virus (127). Several additional studies have found similar associations. Women with BV or CST-IV CVMs were found to have a significantly higher likelihood of persistent HPV infections (127, 128). *Gardnerella vaginalis*, a prominent BVAB, was identified in a prospective longitudinal study by Usyk and colleagues as a biomarker of HPV persistence and neoplastic progression (129). Many of these same studies also found associations between the CVM and HPV clearance. Generally, women with *Lactobacillus*-dominant CVMs classified as CST-I or CST-II had reduced risk for HPV persistence and were more likely to clear HPV infections compared to women with highly diverse CVMs or those with active BV infections (127–130). Furthermore, longitudinal tracking of women found that *Lactobacillus gasseri*-dominated CVMs (CST-II) were associated with the fastest rates of HPV clearance, while highly diverse CVMs (CST-IV) were the slowest (114). Collectively, these studies strongly suggest that BV and cervicovaginal dysbiosis are conducive to increased persistence and reduced clearance of HPV.

However, some studies contradict the link between BV and HPV persistence. A study by Watts et al. found no association between BV and HPV persistence (117). Moscicki and colleagues did not detect significant alterations in CVM composition during HPV persistence (116). Intriguingly, this study also found that dysbiosis and, more specifically, *Gardnerella vaginalis* were associated with a pro-inflammatory “burst” immediately following HPV16 clearance. An additional study also found an association between *Gardnerella vaginalis* and HPV clearance (131). These results suggest that HPV persistence and clearance may be linked to local inflammatory conditions or highly unstable/transient CVM states. Regardless, there is a clear need for additional studies to determine the role of the CVM in shaping the outcome of HPV infection.

### Neoplastic progression and cancer development

The composition of the CVM is also linked to HPV-associated neoplastic disease and cancer development. Mitra and colleagues evaluated the microbial community composition in women with low-grade or high-grade dysplasia or cancer, all of which were verified by histopathological analysis of biopsies (132). With increasing disease severity, they measured a concomitant decrease in *Lactobacillus* abundance and increase in microbiota diversity (CST-IV). Overall, women with *Lactobacillus-*depleted and highly diverse microbial community structures had an approximately twofold increase in low-grade dysplasia, threefold increase in high-grade dysplasia, and fourfold increase in invasive cancer compared to HPV-negative women with no disease. Similar associations between high cervicovaginal microbial diversity and/or *Lactobacillus* depletion and progression to high-grade CIN lesions and/or cancer have been reported (118, 119, 129, 130, 133–136). Consistent with their links to HPV infection and disease, BV and BVAB that often typify highly diverse CVMs are strongly linked to HPV-associated disease progression and cancer (120, 130, 137). Indeed, a study by Oh et al. found that an increase in *Fannyhessea vaginae* and *Gardnerella vaginalis,* two prominent BVAB, and a decrease in *Lactobacillus crispatus* correlated with a significantly higher risk of neoplastic disease (135). Furthermore, *Sneathia* spp. and *Gardnerella vaginalis* correlate with the presence of neoplastic disease and progression to high-grade CIN, respectively, in HPV-infected women (129, 136). Conversely, women with low-diversity CVMs dominated by *Lactobacillus* spp. are more likely to have faster disease regression compared to women with highly diverse, BV-associated CVMs (130). Therefore, BV-associated and other highly diverse CVMs are likely risks for HPV-induced neoplastic disease and progression, whereas *Lactobacillus*-dominated microbial communities are protective.

Alternatively, some studies find no association between BV and neoplastic disease and cancer (117, 138). Despite the strong evidence that *Lactobacillus* dominance in the CVM is protective across stages of infection and disease, the species *Lactobacillus iners* may be an exception. Cervicovaginal *Lactobacillus iners* is significantly associated with high-risk HPV infection and subsequent progression to disease (119, 134, 135) and has been linked to severe dysplasia, perhaps through its link to CST-IV conditions (132, 138). In a recent study by Colbert et al., *Lactobacillus iners* was detected in cervical cancer tumors and its presence linked to treatment responses and intratumoral metabolic changes (139). Discrepancies across clinical studies, as well as the role of intratumoral microbiota in HPV-associated cancer, reinforce the need for mechanistic studies in controlled settings.

## REMAINING QUESTIONS AND POTENTIAL MECHANISMS OF MICROBIOTA EFFECTS ON HPV PATHOGENESIS

There is strong epidemiological and clinical evidence supporting a role for the CVM in regulating aspects of HPV infection and disease progression. However, there are still many remaining questions about the mechanisms and the precise temporal and pathophysiological dynamics underlying this interaction (Box 1). Several emerging mechanistic hypotheses may begin to define how and when the host CVM affects HPV pathogenesis (140, 141) (Fig. 2). The possibilities discussed in this section are rooted in the emerging consensus that *Lactobacillus*-dominant CVMs are protective and those characterized by high diversity and BV-associated bacterial species increase the risk for HPV infection and disease. However, it is worth noting that ~25% of asymptomatic women do not have *Lactobacillus*-dominant CVMs, suggesting there are likely nuances within this relationship to consider (64, 84).

Box 1. **Outstanding questions about the relationship between the host cervicovaginal microbiome and HPV pathogenesis and disease**

How do *Lactobacillus* species protect against HPV infection, persistence, and disease?

How does cervicovaginal dysbiosis facilitate HPV infection?

When and how does the CVM influence HPV-associated disease development and progression?

What molecular events during the HPV life cycle and progression to cancer are influenced by the CVM?

Does HPV infection induce changes in CVM composition that potentiate disease development?

Are the microbiota associated with risk for HPV infection sexually transmitted?

Does the CVM interact differently with low-risk versus high-risk HPVs?

Are there microbial niches within tissues that correlate with HPV risk or protection?

Are the effects of the CVM on HPV pathogenesis direct or indirect?

Do specific intratumoral microbiota contribute to disease progression and/or regression?

### How do *Lactobacillus* species protect against HPV infection, persistence, and disease?

*Lactobacillus* spp. generate conditions favorable for optimal cervicovaginal health and homeostasis. Lactobacilli produce lactic acid, which helps maintain an acidic environment in the cervicovaginal tract. In doing so, *Lactobacillus* spp. prevent colonization and growth of anaerobic bacterial species associated with BV and CST-IV CVMs (142–144). In human cervical organoids, *Lactobacillus*-derived lactic acid regulates cervical stem cell renewal and inhibits HPV E6 and E7-driven organoid growth and progression (145). *Lactobacillus* spp. have also been shown to neutralize potentially carcinogenic byproducts produced by BVAB (146–148). Through the production of antimicrobial peptides and biosurfactants, *Lactobacillus* spp. are critical in maintaining the protective mucus barrier of the cervicovaginal epithelium, which can help limit adherence of and infection with pathogenic bacteria and STIs (149–152). *Lactobacillus* spp. have anti-inflammatory effects and influence immunometabolic pathways that help maintain cervicovaginal homeostasis (144, 153). These mechanisms may help explain how the pathophysiology of CVMs dominated by lactobacilli helps limit HPV infection and neoplastic progression.

### How does cervicovaginal dysbiosis facilitate HPV infection?

In *Lactobacillus-*depleted CVMs, the environment is altered in ways that can increase susceptibility to HPV infection and potentiate neoplastic progression. BVAB produce virulence factors during dysbiosis that alter epithelial barrier functions (85). For example, *Gardnerella vaginalis* secretes sialidases that break down components of the vaginal mucus, thus reducing the fidelity of the protective mucus layer of the cervicovaginal epithelium and causing other disruptions to the CVM (154–156). In a 3D cervical epithelium model, colonization with BVAB species elicited alterations in barrier proteins such as mucin and sialic acid, providing further evidence that bacterial byproducts can alter barrier integrity (153). Mucin degradation can differ across CVM compositions (157). It is possible that sialidase-mediated thinning or loss of the cervicovaginal mucus barrier in certain CVMs exposes the epithelium to a milieu that causes microwounds, which then increase the ability of HPV particles to access basal cells for infection. Interestingly, mucin-degrading enzymes were found to increase specifically in women infected with high-risk HPV16 and HPV18, which perhaps underlies their aggressive attributes and strong links to cancer (124). Vaginolysin is another toxin secreted by *Gardnerella vaginalis* that causes damage and exfoliation of epithelial cells, facilitates bacterial attachment to the epithelium, and is hypothesized to initiate formation of a polymicrobial biofilm (158–160), all of which could act to support HPV infection. Recently, a study found that genetic polymorphisms in genes encoding syndecans, which are heparan sulfate proteoglycans on cervicovaginal epithelial cells, are linked to predisposition to BV (161). Notably, heparan sulfate proteoglycans are involved in mucosal HPV entry into cells (162), making it possible that certain forms of syndecans facilitate BV and, as a result, directly or indirectly increase HPV infection.

### When and how does the CVM influence HPV-associated disease development and progression?

It remains unclear exactly when and through what mechanisms the CVM exerts influence on disease development and progression/regression. Proposed mechanisms include CVM-mediated inflammation, oxidative stress, and immunometabolic alterations. *In vitro* and clinical studies have identified strong associations between high-diversity CVMs, BV, and a pro-inflammatory host response (84–86, 116, 121, 137, 153, 163). In 3D cervicovaginal epithelium models, colonization with *Fannyhessea vaginae* and other BVAB elicited a pro-inflammatory response, whereas a representative protective bacterium, *Lactobacillus crispatus*, did not (86, 153). This demonstrates that pro-inflammatory effects are likely BV-specific. Furthermore, reports indicate that BV and other HPV risk-associated bacterial species increase oxidative stress (153, 164, 165). It is possible that inflammation and oxidative stress induced by BVAB and high diversity CVMs lead to DNA damage, cellular proliferation, and/or influence expression of the HPV E6 and E7 oncoproteins, resulting in integration and malignant transformation (77, 137).

BV and high-diversity CVMs also likely impact the immune and metabolic landscapes of the cervicovaginal tract. Research by the Herbst-Kralovetz lab using 3D cervicovaginal epithelium models demonstrated that colonization with BVAB including *Fannyhessea vaginae*, *Gardnerella vaginalis*, *Prevotella bivia*, and *Sneathia amnii* alters the expression of barrier-related proteins and changes the immunometabolic landscapes in a manner comparable to clinical BV samples (86, 153). Commensal bacteria also regulate immune pathways in vaginal *in vitro* models (166). Several pathways that regulate the immune response were specifically enriched in HPV16 and HPV18-associated CVMs, suggesting there may be distinct microbial compositions associated with HPV-driven malignant transformation (124). The cervicovaginal metabolome is also distinct in HPV-positive women with CST-III versus CST-IV microbial compositions, with different concentrations of amines, glycogen, and phospholipid-related metabolites, among others (167). These dysregulated immune and metabolic pathways may contribute to neoplastic disease progression.

### What molecular events during the HPV life cycle and progression to cancer are influenced by the CVM?

The ways in which the host CVM impacts the molecular mechanisms of the HPV life cycle are largely unclear, particularly whether effects are direct or indirect, global or local, or a combination. Microbiota may influence HPV entry by altering the concentration or form of heparan sulfate molecules available for virion attachment and/or the signaling events triggered by contact between HPV virions and keratinocytes that help initiate infection (14). It is conceivable that the CVM regulates the terminal differentiation process of the stratified epithelium, impinging on the HPV productive life cycle and progeny virion production. The CVM could also regulate viral load following infection, as this is a key determinant of HPV transmission, persistence, and disease progression (34, 39, 40, 168), all aspects of the HPV life cycle that have been linked to CVM composition.

CVMs linked to HPV pathogenesis are often concomitant with inflammation and oxidative stress that can potentiate DNA damage (137, 169). Cellular DNA damage and stress response pathways are required for episome maintenance and viral genome amplification (15, 16, 170–172), so one can envision how CVM-induced alterations to DNA damage and/or repair mechanisms may influence and perhaps exacerbate the HPV productive life cycle. Furthermore, DNA repair factors are preferentially recruited to HPV genomes at the expense of host DNA repair (107, 171, 173). This vulnerable genomic state, combined with CVM-induced inflammation and oxidative stress, which is known to increase HPV integration in keratinocytes (174), may potentiate genomic instability and viral genome integration. Interestingly, the cervicovaginal epithelial epigenome can correlate with microbial community composition (175, 176) and HPV integration sites in host DNA are controlled by epigenetic regulation (177, 178). Therefore, it is possible that the CVM influences epigenetic regulation on both host and viral DNA in ways that potentiate infection and cancer development. How the CVM may influence the physical state of the HPV genome has not been widely explored, but integration often results in elevated E6 and E7 and provides cells with a growth advantage (42), thus providing another possible link to HPV pathogenesis.

### Does HPV infection induce changes in CVM composition that potentiate disease development?

A fundamental question that remains unanswered is whether microbial communities present at the time of infection dictate the outcome of HPV infection, or if CVM changes are secondary to HPV infection and then drive viral persistence and disease. In women, HPV infection has been reported to be a key determinant of CVM bacterial diversity and dominates the virome when present (179). Women who achieve high viral loads during infection with high-risk HPV16 and HPV18, but not other HPV types, were less likely to have a CVM dominated by *Lactobacillus crispatus* (CST-I), which the authors proposed could be due to type-specific persistence and clearance dynamics (124). Similarly, different CVM compositions were measured in precancerous lesions in women infected with HPV16 versus HPV52 (180). A recent study by Bridy et al. reported that cervicovaginal epithelial cells expressing episomal HPV16 genomes responded differently to colonization with *Sneathia vaginalis* in 3D raft models compared to HPV-negative cells, supporting more bacterial growth and exhibiting increased resistance to bacterial cytotoxicity (181). In animal models, epithelial expression of the HPV oncoproteins alone can alter the microbiome (182), and murine papillomavirus infection, viral load, persistence, and disease state all significantly influence the murine CVM (183). This evidence suggests that HPV infection influences the CVM while simultaneously shaping the spatiotemporal growth dynamics of microbiota, likely implicating a bidirectional relationship.

## CHALLENGES AND SOLUTIONS FOR STUDYING HPV AND THE MICROBIOME

### Challenges studying HPV and the microbiome

There are several remaining questions about the relationship between HPV and the host microbiome (Box 1). Clinical studies have and will continue to provide invaluable insight into this association. While paradigms are emerging based on these data, there remain inconsistencies across studies and populations that demand biological explanations. Inherent challenges of studying both papillomaviruses and the microbiome have hindered such mechanistic investigations. The strict species and tissue specificity of papillomaviruses has historically complicated the development of preclinical models that faithfully recapitulate viral infection, transmission, and disease development. Microbiome studies are often plagued by species-specific microbiota, tissue-specific growth requirements, and high variability. Furthermore, humans are unique in having *Lactobacillus*-dominant CVMs under healthy conditions, a trait that is not found in common laboratory animals like rodents, non-human primates, and other mammals (184). Despite these challenges, there are now established and/or emerging preclinical models to help close several gaps in knowledge and complement ongoing clinical studies.

### Established and emerging preclinical models to study HPV and the microbiome

Since the discovery that HPVs are causal agents of cervical cancer in the 1980s (24), there has been extraordinary progress in our understanding of the HPV life cycle and its relationship to the host. Much of this progress has been a result of the advancement of preclinical models. For example, the introduction of HPV viral genomes into keratinocytes cultured in 3D stratified epithelium equivalents, also known as organotypic raft cultures, recapitulates the complete HPV life cycle and produces progeny virions *in vitro* (185–187). These 3D models are generally composed of a dermal equivalent containing collagen-embedded fibroblasts and keratinocytes layered at an air–liquid interface, which differentiate and closely mimic a stratified squamous epithelium (188). Similar *in vitro* 3D models have been developed to study bacterial colonization and subsequent immune, metabolic, structural, and other responses in female reproductive tissue equivalents (86, 87, 153, 166, 189, 190). These models can be adapted to study interactions between HPV-positive epithelial cells, microbiota, and other microbiome components using keratinocytes derived from multiple anatomical sites to better mimic site-specific conditions (191). The Chumduri lab has developed 3D organoids using patient-derived cervical epithelial cells genetically modified to express HPV oncoproteins to model and investigate HPV-chlamydia co-infections (192, 193). Additional 2D models using bacterial cell-free extracts can be used to investigate CVM components in human cells under various conditions (139, 190). By combining elements from HPV replication and bacterial colonization models, these evolving and increasingly complex *in vitro* systems are important platforms to help define how the CVM influences HPV infection and the viral life cycle.

Although *in vitro* models are incredibly valuable tools, they do not fully recapitulate the biological intricacies of a living organism. The strict species specificity of papillomaviruses has made studying HPV pathogenesis in laboratory animals challenging, as animals do not support productive HPV infections. Thus, researchers have used two main *in vivo* approaches to circumvent this issue: transgenic mice and animal papillomavirus models (194). Laboratory mice, or *Mus musculus,* are an attractive choice for preclinical models as they are the most well-characterized and technically supported animal used in biomedical research (195). HPV transgenic mice genetically engineered to express the high-risk HPV16 viral proteins have provided significant insight into HPV-mediated neoplastic disease progression and carcinogenesis (196). Several types of HPV transgenic mice are available that express either the entire early region of the HPV16 viral genome (197), or the individual oncoproteins E5 (198), E6 (199), or E7 (199). In these mice, HPV protein expression is normally targeted to the basal cells of the stratified epithelium by a keratin 14 (K14) promoter, and there are now strains of mice with constitutive, inducible, and/or conditional expression of the HPV oncoproteins (196, 200, 201). The Herfs group recently used *K14-HPV16* mice to explore the effect of HPV proteins and disease on the murine CVM (182). In the squamous epithelium of HPV transgenic mice, they found reduced expression of host antimicrobial peptides that are used by *Lactobacillus* spp. as an energy source. Moreover, the CVM of *K14-HPV16* mice differed in those that developed low-grade versus high-grade dysplasia, findings that were consistent with another study in *K14-E7* transgenic mice (202). *Lactobacillus* spp. abundance also decreased as disease severity increased. These results indicate that HPV oncoprotein-induced neoplastic disease correlates with changes in the murine CVM.

Notably, HPV transgenic mice do not model viral infection, persistence, integration, and the development of subsequent neoplastic disease that occurs because of infection. Until the discovery of a murine papillomavirus, MmuPV1, in 2011 (203), HPV researchers lacked an infection-based murine model to study papillomaviruses. However, MmuPV1 infection models are now transforming the capacity to address outstanding questions that have heretofore been difficult to investigate in preclinical animal models (183). MmuPV1 exhibits dual tropism and can infect cutaneous sites including the tail, ears, and dorsal skin as well as mucosal epithelia of the cervix, vagina, penis, anus, oral cavity, and vocal folds (203–219). Although there are genetic and biochemical differences between MmuPV1 and high-risk HPVs, MmuPV1 productively infects murine epithelia, establishes long-term persistent infections, and causes progressive neoplastic disease and cancer (220–222). Furthermore, we discovered that MmuPV1 is sexually transmitted, thus establishing the first murine model of papillomavirus sexual transmission (212).

We recently used the MmuPV1 cervicovaginal infection model in immunocompetent mice to determine the effects of papillomavirus infection on the murine CVM (183). We discovered that key aspects of MmuPV1 infection, such as viral load, stage of infection, and persistence/clearance, all significantly altered the CVM composition in MmuPV1-infected mice across multiple experiments. Infection also caused significantly larger shifts in microbial community compositions over time compared to mock-infected mice. Consistent with the study by Lebeau et al. in HPV16 transgenic mice (182), severity of disease in MmuPV1-infected mice also influenced the murine CVM. Using laser-capture microdissection to analyze microbial composition within specific disease stages, we discovered that disease severity also influences the composition of the intralesional and intratumoral local microbiome. MmuPV1 models provide powerful new opportunities to investigate the host microbiome and papillomavirus pathogenesis.

Recent advances in murine models of BV and other approaches to manipulate the CVM in mice provide additional tools to study HPV-CVM interactions. Gilbert and colleagues recently colonized mice with *Gardnerella vaginalis* and *Prevotella bivia*, which recapitulated several aspects of human BV (223). Systemic antibiotic treatment can also be used to induce dysbiosis in mice (224). The Patras group recently reported that gavaging female mice with human fecal samples surprisingly results in a ‘humanized’ *Lactobacillus-*dominant CVM (225). In the future, these preclinical models of viral pathogenesis and bacterial colonization can be adapted for use in germ-free or gnotobiotic mice to specifically define the causal and temporal effects of single bacterial species and polymicrobial communities on viral infection and disease. Emerging models, together with clinical studies and more modernized computational approaches (140), are key to defining the mechanisms that underlie the bidirectional interactions between HPV and the CVM and how they impact cervicovaginal health and disease.

## CONCLUSIONS AND FUTURE PERSPECTIVES

Several lines of evidence point to a strong relationship between the host microbiome and HPV across all stages of infection and disease. However, our understanding of the mechanisms involved in this interaction remains woefully limited, mainly due to technical challenges inherent to both HPV and microbiome research. However, emerging models offer promising new opportunities to longitudinally study the spatiotemporal dynamics of HPV-CVM interactions. The microbiome is important to the pathogenesis of several viruses (94), and the approaches used to study these virus–microbiota interactions may prove instructive for the investigations of HPV and the CVM.

By coupling new and physiologically relevant *in vitro* and *in vivo* models with well-designed clinical studies, we are poised to gain tremendous insight into the bidirectional relationship between HPV and the host microbiome. These tractable preclinical models will provide much-needed platforms to help discern between direct versus indirect mechanisms of the CVM on HPV pathogenesis, as well as targeted investigations of issues such as anatomical microbiota niches, hormonal influences, intratumoral microbiota, sex-specific communities, and sexual transmission of microbiota. These opportunities extend beyond the cervicovaginal tract to other anatomical sites infected by HPVs as there is evidence that the microbiomes of the oral, anal, penile, and skin epithelia associate with HPV infection and pathogenesis (226–230). Likewise, the impact of the microbiome on low-risk and cutaneous HPVs can also be explored. As models continue to evolve in complexity and precision, the roles of other microbiota reported to have a role in HPV pathogenesis, such as fungi and other viruses (59–61, 129, 231, 232), should also be explored. As our understanding of causal relationships between microbiota and HPV pathogenesis improves, there is immense therapeutic potential to design interventions that prevent and/or treat HPV infection, persistence, and disease (169). In conclusion, viruses encounter and replicate in an environment full of microorganisms. When we consider and attempt to mimic these conditions, there is likely an entirely new realm of discovery possible that will impact our understanding of viral pathogenesis, disease, and its treatment.
